# Creating a longitudinal dataset of care experienced children in Scotland – Administrative Data Research Scotland.

**DOI:** 10.23889/ijpds.v7i3.2009

**Published:** 2022-08-25

**Authors:** Cecilia Macintyre, Gillian Raab

**Affiliations:** 1 Scottish Government; 2 University of Edinburgh

To create a dataset which describes the care experience of children in Scotland data. To share with analysts in a safe setting to allow linkage, and provide information on the strengths and weaknesses. To gather feedback on its use to inform future collections and improve experience of future users.

The dataset was created by combining 11 years of data provided to Scottish Government by local authorities. The resulting longitudinal dataset includes details of the children, the periods of care including the type of care setting, along with the legal basis for this care, and information on the destination of the child following care. Detailed information about the dataset is provided along with a background document in how the dataset was constructed. Data quality flags are provided to highlight situations where there may be inconsistences, and code is shared to create a cleaned dataset for analysis.

A longitudinal dataset has been created covering the period 2009 to 2019. This covers almost 60,000 children with details of the care setting and legal basis. The dataset has been indexed to a population spine, which enables it to be linked to other data. Additional derived variables have been added, and improvements made to data quality where possible. In other situations data quality is highlighted with guidance provided on how to deal with these issues. The results have been shared extensively with the data providers, and previous users providing valuable input. This has resulted in improved understanding of the data, and informed the practice of gathering data in future years, and plans for official statistics on health outcomes of care experienced children.

This project has created an enduring resource which will improve evidence for policy making by allowing analysis to consider patterns of care experience and link it to outcomes. This is particularly relevant given a review of care experience in Scotland, and the commitment by Scottish Government to keep the Promise.

